# Association of motoric cognitive risk syndrome and incident mild cognitive impairment in community-dwelling older adults

**DOI:** 10.3389/fneur.2026.1787145

**Published:** 2026-03-20

**Authors:** Ying Zhang, Lixin Ma, Yanjun Ma, Jie Chang, Yiwei Zhao, Xue Gao, Yue Wu, Yiwen Xing, Yansu Guo, Lina Ma, Zhibin Wang, Yi Tang

**Affiliations:** 1 Department of Neurology, The First Hospital of Hebei Medical University, Shijiazhuang, Hebei, China; 2 Department of Neurology, Handan Central Hospital, Handan, Hebei, China; 3 National Center for Neurological Disorders, Xuanwu Hospital, Capital Medical University, Beijing, China; 4 Department of Neurology & Innovation Center for Neurological Disorders, Xuanwu Hospital, Capital Medical University, National Center for Neurological Disorders, Beijing, China; 5 Department of Neurology, Beijing Fengtai You'anmen Hospital, Beijing, China; 6 Beijing Geriatric Healthcare Center, Xuanwu Hospital, Capital Medical University, Beijing, China; 7 Department of Geriatrics, Xuanwu Hospital, Capital Medical University, Beijing, China; 8 Neurodegenerative Laboratory of Ministry of Education of the Peoples Republic of China, Beijing, China

**Keywords:** cognitive decline, community-dwelling older adults, gait speed, mild cognitive impairment, motoric cognitive risk syndrome, subjective cognitive complaint

## Abstract

**Background:**

Motoric cognitive risk syndrome (MCR) has been recognized as a risk factor for dementia, but its role in the transition to mild cognitive impairment (MCI) among community-dwelling older adults remains unclear. This study investigated the associations between MCR and the risk of incident MCI or cognitive decline over a one-year follow-up period.

**Methods:**

We investigated whether MCR predicted the risk of incident MCI and cognitive decline (Mini-Mental State Examination decline ≥ 4 points) in 853 community-dwelling older adults aged 60 years and above from a northern Chinese urban area, all without dementia or disability at baseline. Logistic regression, adjusted for potential confounders, was used to assess the overall prediction value of MCR and the associations of its cognitive (subjective cognitive complaint, SCC) and motoric (gait speed) components with the risk of MCI.

**Results:**

Participants with MCR had a higher risk of developing MCI [adjusted odds ratio (OR) = 2.22, 95% confidence interval (CI): 1.18–4.17] and cognitive decline (adjusted OR = 2.28, 95% CI: 1.11–4.70). Among the individual components of MCR, slow gait speed was significantly associated with MCI risk (adjusted OR = 2.15, 95% CI: 1.19–3.87). Subgroup analysis in the MCR population showed that individuals under 75 years of age and women were at greater risk of incident MCI.

**Conclusion:**

MCR, characterized by subjective cognitive complaints and slow gait speed, is significantly associated with an increased risk of developing MCI and cognitive decline in this cohort of community-dwelling older adults in northern China.

## 1 Introduction

By mid-century, the number of people living with dementia is projected to reach 152.8 million (1), with associated costs expected to rise to $9.12 trillion worldwide (2). Given the lack of curative treatments and the high disability burden of dementia (3), early identification and risk management are critical. As the global population ages, concurrent cognitive and motor function declines have been widely observed in the elderly (4–7). This phenomenon, known as motoric cognitive risk syndrome (MCR), is characterized by the co-occurrence of subjective cognitive complaint (SCC) and slow gait speed in individuals without dementia or mobility disability (8). The prevalence of MCR among elderly people is approximately 10% based on worldwide population-based studies (9).

Cohort studies have established a link between MCR and an increased risk of dementia and cognitive impairment, highlighting its role in accelerating cognitive decline (8, 10–13). This predictive power positions MCR as a key indicator within the spectrum of pre-dementia risk states (14), alongside the well-established syndrome of mild cognitive impairment (MCI), which is characterized by objective cognitive deficits without functional impairment (15–17). While MCI and MCR are two subtypes of pre-dementia states, they are not mutually exclusive. Substantial overlap exists, with approximately 40% of individuals meeting MCR criteria also meeting criteria for MCI (12). Despite this demonstrated overlap and their shared predictive power for future cognitive decline, it remains unclear whether MCR accelerates the transition to MCI.

In this study, we investigated the predictive value of MCR for incident MCI and cognitive decline over a one-year follow-up period in a distinct cohort of community-dwelling older adults from northern China. We specifically examined MCR as an integrated measure, analyzed the independent roles of SCC and gait speed, and conducted subgroup analyses to provide a granular understanding within this specific demographic and geographical setting. Our findings highlight a clear relationship between MCR and longitudinal decline in cognitive function with advancing cognitive stages in MCI, suggesting its potential as a monitoring and intervention target for dementia prevention.

## 2 Methods

### 2.1 Study design

We conducted a prospective cohort study from May 2023 to October 2024 as part of the Beijing Disability Risk and Ageing Monitoring (BEAM) study (ClinicalTrials.gov identifier: NCT 06394817). The BEAM study is an ongoing cohort study designed to assess disability risk among older adults in the Baizhifang community in Beijing, China. For the current analysis focusing on the relationship between MCR and MCI, a 1-year follow-up period was employed. This duration was pragmatically selected to capture clinically meaningful cognitive transitions within a timeframe relevant to early intervention strategies. All data were collected through face-to-face interviews. Before enrollment, written informed consent was obtained from all participants, and the study protocol was approved by the Ethics Committee of Xuanwu Hospital, Capital Medical University (2022–234). This study adhered to the Strengthening the Reporting of Observational Studies in Epidemiology (STROBE) guidelines for cohort studies.

### 2.2 Participants

The BEAM study included Chinese adults aged 60 years or older who had lived in the target community for at least 1 year before the survey date. Individuals with inadequate hearing or vision were excluded. A total of 2,018 participants were initially enrolled, with 1,622 (80.4%) retained after 1 year to assess disability as the primary outcome.

**Figure 1:**
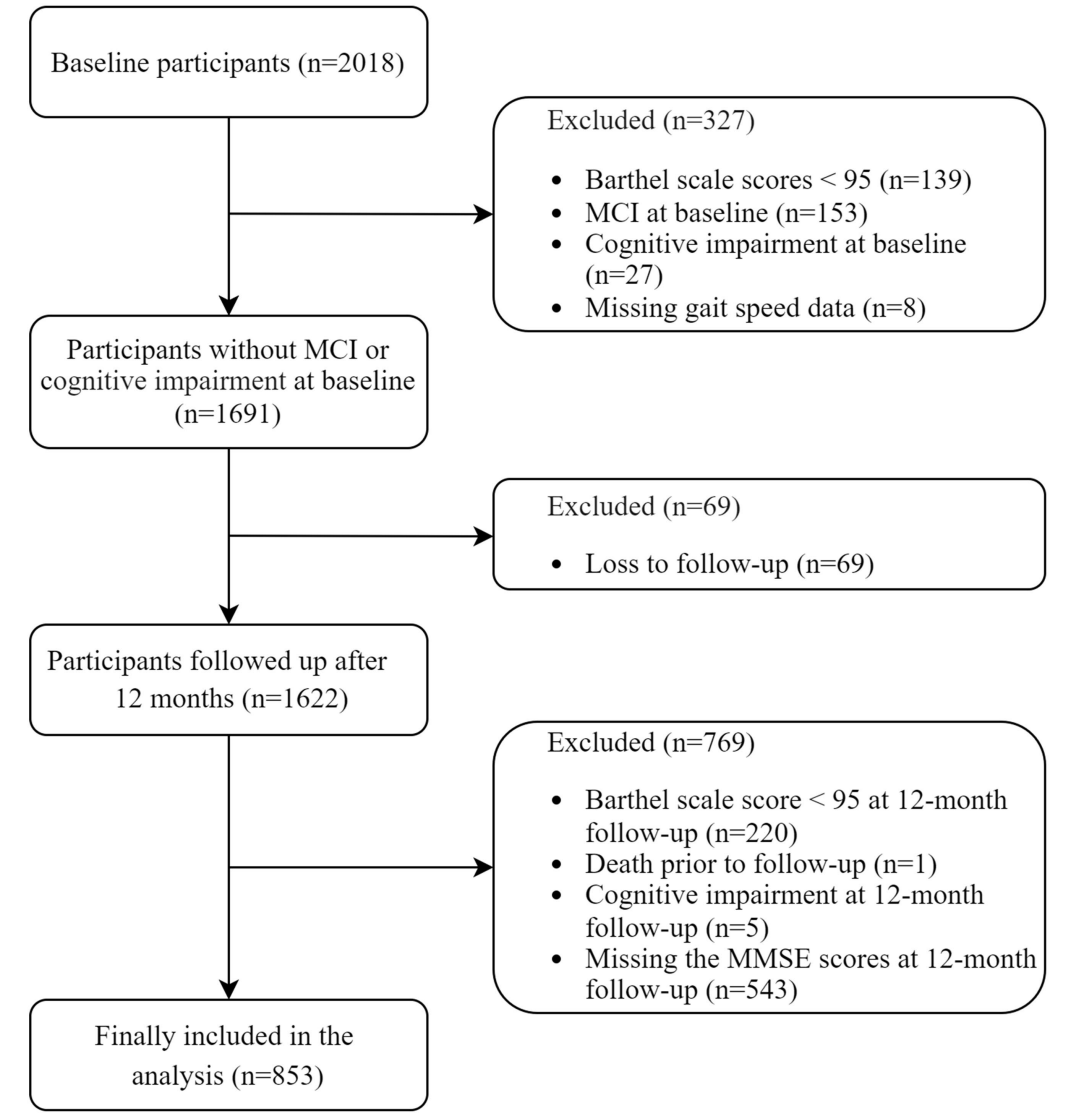
Study flow diagram. MCI, mild cognitive impairment; MMSE, mini-mental state examination. Cognitive impairment was defined by MMSE cut-off scores of < 17 for illiterate individuals, < 20 for those with elementary school education, and < 24 for those with middle school education or higher.

To isolate the predictive role of MCR for incident MCI, participants with baseline MCI (*n* = 153) — among whom 19 (12%) also met MCR criteria — were excluded due to clinical overlap and to ensure a baseline sample free of objective cognitive impairment. Further exclusions at baseline included individuals with Barthel scale scores below 95 (*n* = 139), missing gait speed data (*n* = 8), or other cognitive impairment (*n* = 27). Additionally, participants lost to follow-up (*n* = 69) or with incomplete data at the 1-year follow-up (*n* = 769) — defined as missing key outcome variables necessary for assessing cognitive status or functional independence — were excluded. After applying these exclusions, 853 participants were included in the final analysis (Figure 1).

### 2.3 MCR

MCR was diagnosed based on established criteria, requiring the simultaneous presence of SCC and slow gait speed in the absence of dementia or mobility disability (8) (Supplementary Figure 1).

#### 2.3.1 Subjective cognitive complaint (SCC)

SCC was assessed using standardized questionnaires adapted from the Geriatric Depression Scale (GDS) (18) and Clinical pHysical rEsilience assEssment Scale (CHEES) (19). Participants were categorized as having SCC if they responded “yes,” “quite agree,” or “agree” to either of the following questions:

1. “Do you feel you have more problems with memory than most people?”
2. “Do you increasingly find it difficult to remember where you put things?”

#### 2.3.2 Slow gait speed

Participants completed two 4-meter walks at their usual pace from a standing start, with at least 1 meter beyond the course to prevent deceleration. The mean of both trials was used for analysis. Slow gait speed was defined as a walking speed at least one standard deviation below the age- and sex-specific means for our cohort, a criterion commonly employed and validated in the literature for MCR diagnosis (8, 20) (Supplementary Table 1).

### 2.4 Functional independence and dementia criteria

Functional independence was classified as a Barthel scale score ≥ 95 (21). Dementia was diagnosed based on the Diagnostic and Statistical Manual of Mental Disorders, Fifth Edition (DSM-5) (22) or a Mini-Mental State Examination (MMSE) score ≤ 13 (23).

### 2.5 Cognitive outcomes

The primary outcome, MCI, was diagnosed using the modified Mayo Clinic criteria (17, 24). MCI was defined as objective cognitive impairment, relatively preserved general cognitive function, functional independence, and the absence of dementia (Supplementary Figure 1).

Objective cognitive impairment was defined as an MMSE score at least one standard deviation below the mean for education-matched individuals in our cohort (25) (Supplementary Table 2). Based on established MMSE cut-offs for the Chinese population (26), general cognitive function was considered relatively preserved: 17 for illiterate individuals, 20 for those with elementary school education, and 24 for those with middle school education or higher.

Cognitive decline was defined as a reduction of ≥ 4 points in MMSE scores during follow-up (12, 27). This threshold is recognized as a reliable indicator of minimal clinically important difference (MCID) in longitudinal studies of older adults (28). For example, a drop from 26 to 22 may reflect significant difficulties in recalling recent events, orienting to time or place, or performing simple calculations—tasks essential for daily functioning (25). Such a decline, even without meeting formal MCI criteria, signals a meaningful shift in cognitive capacity and is associated with increased risk of progression to dementia.

### 2.6 Covariates

The covariates in this study included gender, age, educational level, alcohol consumption, smoking status, body mass index (BMI), hypertension, diabetes mellitus, dyslipidemia, coronary heart disease, and stroke. Education was divided four levels: elementary school or below, middle school, high school, and college or above. BMI was categorized into four levels (29): underweight (< 18.5 kg/m^2^), normal weight (18.5 ≤ BMI < 24 kg/m^2^), overweight (24 ≤ BMI < 28 kg/m^2^), and obesity (≥ 28 kg/m^2^). Participants were classified as having medical comorbidities if they had a physician-diagnosed condition or were undergoing pharmaceutical treatment for the disease (Supplementary Table 3).

### 2.7 Statistical analysis

The baseline characteristics of participants with and without MCR were compared using independent *t*-tests for continuous variables and chi-square tests for categorical variables. Logistic regression models were used to examine the association between MCR and the risk of MCI or cognitive decline, adjusting for age, gender, educational level, current smoking and drinking status, BMI, hypertension, diabetes mellitus, dyslipidemia, coronary heart disease, and stroke.

Subgroup analyses were conducted to explore potential variations using the same adjustments. Results were reported as odds ratios (OR) with 95% confidence intervals (CI). All statistical tests were 2-sided, with *p* < 0.05 considered statistically significant. All analyses were performed using SPSS (Version 27, IBM Corp.).

## 3 Results

### 3.1 Participants characteristics

The overall prevalence of MCR among individuals aged 60 years and older was 15.0% (95% CI: 12.7–17.6%). Detailed prevalence rates of MCR stratified by age, sex, and education are provided in Supplementary Table 4.

At baseline, 128 participants met the MCR diagnostic criteria, with ages ranging from 60 to 85 years (mean: 69.1 years). Among them, 47 (36.7%) were men and 81 (63.3%) were women.

Table 1 compares the baseline characteristics of MCR and non-MCR participants. There were no significant differences in age, sex, educational level, MMSE scores, smoking status, alcohol consumption, or BMI. The prevalence of hypertension, diabetes mellitus, dyslipidemia, and stroke were also comparable between the two groups. However, the MCR group had a lower prevalence of coronary heart disease than the non-MCR group (14.8% *vs*. 23.9%, *p* = 0.024).

**Table 1:** Demographic and clinical characteristics of the participants at baseline.

Variables	Total (*n* = 853)	MCR (*n* = 128)	Non-MCR (*n* = 725)	*p-*value
Sex, *n* (%)	–	–	–	0.971
Male	312 (36.6)	47 (36.7)	265 (36.6)	–
Female	541 (63.4)	81 (63.3)	460 (63.4)	–
Age (years), Mean (SD)	69.1 (5.3)	69.3 (5.2)	69.0 (5.3)	0.588
Education, *n* (%)	–	–	–	0.311
Elementary school or below	16 (1.9)	2 (1.6)	14 (1.9)	–
Middle school	227 (26.6)	26 (20.3)	201 (27.7)	–
High school	314 (36.8)	54 (42.2)	260 (35.9)	–
College or above	296 (34.7)	46 (35.9)	250 (34.5)	–
Gait speed (cm/s), Mean (SD)	81.1 (18.4)	52.5 (8.4)	85.0 (15.0)	0.001
MMSE, Mean (SD)	29.0 (1.0)	28.8 (1.0)	29.0 (0.9)	0.389
Smoking, *n* (%)	–	–	–	0.665
Current/former	172 (20.2)	24 (18.8)	148 (20.4)	–
Never	681 (79.8)	104 (81.3)	577 (79.6)	–
Alcohol consumption, *n* (%)	–	–	–	0.294
Current/former	177 (20.8)	31 (24.2)	146 (20.1)	–
Never	676 (79.2)	97 (75.8)	579 (79.9)	–
BMI (kg/m^2^), *n* (%)	–	–	–	0.595
< 18.5	35 (4.1)	6 (4.7)	29 (4.0)	–
18.5–23.9	333 (39.0)	44 (34.4)	289 (39.9)	–
24.0–27.9	339 (39.7)	52 (40.6)	287 (39.6)	–
≥ 28	146 (17.1)	26 (20.3)	120 (16.6)	–
Hypertension, *n* (%)	496 (58.1)	79 (61.7)	417 (57.5)	0.374
Diabetes mellitus, *n* (%)	235 (27.5)	37 (28.9)	198 (27.3)	0.709
Dyslipidemia, *n* (%)	457 (53.6)	69 (53.9)	388 (53.5)	0.935
Coronary heart disease, *n* (%)	192 (22.5)	19 (14.8)	173 (23.9)	0.024
Stroke, *n* (%)	120 (14.1)	15 (11.7)	105 (14.5)	0.407

BMI, body mass index (calculated as weight in kilograms divided by height in meters squared); cm, centimeter; kg, kilograms; m, meter; MCR, motoric cognitive risk syndrome; MMSE, mini-mental state examination; s, second.

Continuous data are presented as mean (standard deviation) or median (interquartile range), and categorical variables as numbers (percentage).

Differences were assessed using Chi-Square Test or *T*-Test.

Self-report of physician diagnosis of hypertension, diabetes, dyslipidemia, coronary heart disease, and stroke or on pharmaceutical treatment for these diseases.

### 3.2 Associations between MCR and risk of MCI or cognitive decline

During the one-year follow-up, 16 of 128 participants (12.5%) in the MCR group developed incident MCI, compared to 45 of 725 subjects (6.2%) in the non-MCR group (risk ratio = 2.02, 95% CI: 1.10–3.67). Logistic regression analysis confirmed this association across different models (Table 2). Model 1, which presented unadjusted associations, showed that individuals with MCR had a significantly higher risk of MCI (OR = 2.16, 95% CI: 1.18–3.95), indicating a 116% increased likelihood compared to those without MCR. This association remained significant in Model 2, adjusted for covariates (adjusted OR = 2.22, 95% CI: 1.18–4.17).

**Table 2:** Association of MCR with the risk of MCI and cognitive decline.

Variables		MCI				Cognitive decline			
		Model 1		Model 2		Model 1		Model 2	
		OR (95% CI)	*p-*value	OR (95% CI)	*p-*value	OR (95% CI)	*p-*value	OR (95% CI)	*p-*value
MCR	No (*n* = 725)	(Reference)	–	(Reference)	–	(Reference)	–	(Reference)	–
	Yes (*n* = 128)	2.16 (1.18–3.95)	0.013	2.22 (1.18–4.17)	0.013	2.31 (1.15–4.63)	0.018	2.28 (1.11–4.70)	0.026
SCC	No (*n* = 208)	(Reference)	–	(Reference)	–	(Reference)	–	(Reference)	–
	Yes (*n* = 645)	1.68 (0.84–3.38)	0.145	1.68 (0.82–3.43)	0.158	1.78 (0.61–5.24)	0.293	1.95 (0.65–5.88)	0.234
Slow gait speed	No (*n* = 687)	(Reference)	–	(Reference)	–	(Reference)	–	(Reference)	–
	Yes (*n* = 166)	2.15 (1.22–3.77)	0.008	2.15 (1.19–3.87)	0.011	1.87 (0.80–4.37)	0.151	1.66 (0.68–4.04)	0.268

CI, confidence interval; MCR, motoric cognitive risk syndrome; MCI, mild cognitive impairment; OR, odds ratio; SCC, subjective cognitive complaint.

Cognitive decline was defined as a decline of ≥ 4 points on the Mini-Mental State Examination, a threshold indicating minimal clinically important difference (MCID).

Model 1: Unadjusted.

Model 2: Adjust for age, sex, education, smoking, drinking, body mass index, hypertension, diabetes mellitus, dyslipidemia, coronary heart disease, and stroke.

A separate logistic regression analysis also examined whether MCR was associated with an increased risk of cognitive decline, defined as an MMSE score reduction of ≥ 4 points. As shown in Table 2, participants with MCR had a significantly higher risk of cognitive decline, with an adjusted OR of 2.28 (95% CI: 1.11–4.70).

### 3.3 Associations between SCC, gait speed, and the risk of MCI or cognitive decline

To evaluate the independent contributions of SCC and gait speed, the two components of MCR, we conducted logistic regression analyses to assess their association with the risk of incident MCI or cognitive decline. Gait speed was significantly associated with an increased risk of MCI (adjusted OR = 2.15, 95% CI: 1.19–3.87). SCC, however, was not significantly associated with MCI (adjusted OR = 1.68, 95% CI: 0.82–3.43). When assessing cognitive decline (MMSE decline ≥ 4 points), neither SCC (adjusted OR = 1.95, 95% CI: 0.65–5.58) nor gait speed (adjusted OR = 1.66, 95% CI: 0.68–4.04) showed a significant association (Table 2). These findings suggest that among the MCR components, slow gait speed independently increases the risk of incident MCI, whereas SCC alone does not.

### 3.4 Subgroup analysis

**Figure 2:**
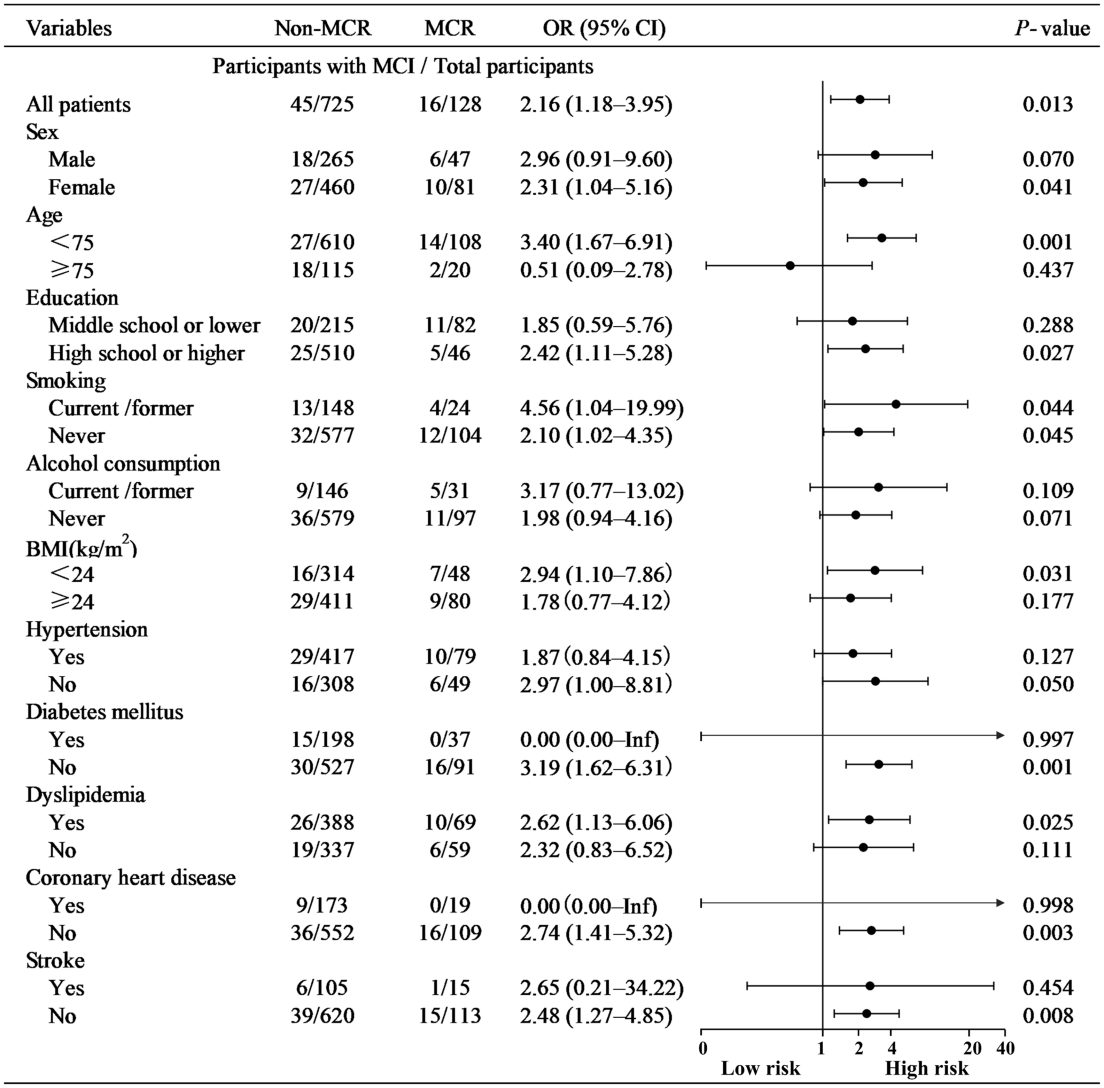
Subgroup analysis based on demographic and clinical characteristics. BMI, body mass index (calculated as weight in kilograms divided by height in meters squared); CI, confidence interval; kg, kilograms; m, meter; MCR, motoric cognitive risk syndrome; MCI, mild cognitive impairment; OR, odds ratio.

A subgroup analysis was conducted to explore variations in the risk of MCI among MCR participants based on age, sex, educational level, BMI, smoking status, alcohol consumption, and comorbidities (Figure 2). While interactions between MCR and subgroup variables were not statistically significant, several trends emerged. Women with MCR had a higher risk of developing MCI than those without MCR (adjusted OR = 2.31, 95% CI: 1.04–5.16). Similarly, participants younger than 75 years with MCR had more than twice the likelihood of transitioning to MCI (adjusted OR = 3.40, 95% CI: 1.67–6.91). Those with a high school education or higher were more likely to progress to MCI (adjusted OR = 2.42, 95% CI: 1.11–5.28). Participants with both MCR and dyslipidemia had a significantly increased risk of MCI (adjusted OR = 2.62, 95% CI: 1.13–6.06). These findings suggest that certain subgroups—particularly younger individuals, women, those with higher education levels, and individuals with dyslipidemia—may be at greater risk of MCI when MCR is present.

## 4 Discussion

This study investigated the association between MCR and the risk of developing MCI or cognitive decline over a one-year follow-up. Our findings indicated that individuals with MCR face a significantly higher risk of transitioning to MCI or cognitive decline, with motor dysfunction emerging as the primary risk factor for this progression. This observation supports the conceptualization of MCR as a predictor to MCI, reflecting an earlier stage of cognitive aging where subjective complaints and motoric impairment may precede objective cognitive decline. To examine this hypothesized directional pathway—from motoric-subjective risk to objective cognitive impairment – our study design excluded all individuals with baseline MCI. This approach allowed us to isolate the predictive role of MCR for incident MCI.

Indeed, individuals with MCR but without baseline MCI exhibited a 2.22-fold increased risk of transitioning to incident MCI within 1 year follow-up in our cohort. This finding underscores the significant predictive power of MCR, clearly demonstrating a temporal sequence and substantial risk magnitude in the progression towards MCI. Such predictive capability aligns with previous research that identified MCR as a risk factor for cognitive decline: a multi-country study reported a 1.90-fold increased risk of developing dementia among individuals with MCR (12), and the Mexican Health and Aging Study found a 2.52-fold higher risk of cognitive impairment in older adults with MCR (30). While MCR and MCI overlap on the dementia risk continuum (12), our results specifically highlight MCR’s role as a significant predictor to incident MCI. These findings reinforced the role of MCR as a transitional stage between normal aging and dementia, providing a window for early identification and intervention (16). However, given the observational nature of our study, causality cannot be established. Future large-scale, multicenter, and longitudinal studies are necessary to determine whether MCR directly contributes to the progression of MCI.

Although prior research has demonstrated that MCR predicts cognitive decline, the relative contributions of cognitive or motor components remain unclear. Some evidence suggests that cognitive impairment may be the primary driver, as one study found that the severity of cognitive deficits, rather than motoric impairments, were stronger predictors of dementia conversion (13). Additionally, up to 45% of older adults with SCC progress to MCI within 4 years, and their dementia risk is twice that of cognitively normal individuals (31, 32). However, our findings support motor dysfunction as a key predictor, as participants with slow gait speed were 2.15 times more likely to develop MCI. This aligns with previous studies demonstrating that slow gait speed is an early marker of cognitive decline, often deteriorating up to 12 years before MCI onset (5, 33–36). In our analysis, SCC demonstrated a weaker predictive association with incident MCI compared to slow gait speed. This may be because gait speed, as an objective measure of physical function, can serve as a more robust upstream marker of neurodegenerative processes that ultimately lead to MCI. Therefore, the motoric component of MCR plays a crucial role in identifying individuals at high risk of MCI and cognitive decline.

The interplay between cognitive and motoric function may stem from shared neural and physiological mechanisms. Both cognition and gait speed rely on overlapping cortical regions and executive function, and they share common risk factors such as cardiovascular disease and diabetes (37–39). Additionally, age-related declines in muscle strength may contribute to both slower gait and cognitive impairment (35, 40, 41), further reinforcing their link. This relationship suggests that MCR—by integrating cognitive and motoric phenotypes—represents a key transitional stage in the aging process (42). The practicality of MCR assessment—requiring only a timed walk and a few questions—makes it ideally suited for primary care and community settings where resources for extensive neuropsychological testing are limited. Our findings suggest that older adults presenting with slowed gait, especially when accompanied by cognitive concerns, should be prioritized for closer monitoring and potential referral. These findings have significant public health implications. The simplicity and low cost of MCR screening allow for scalable implementation across diverse healthcare settings, enabling population-level early risk stratification. By identifying high-risk individuals, healthcare systems can target limited resources more efficiently—focusing intensive assessments and preventive interventions on those most likely to progress to MCI. Early detection also facilitates timely initiation of multimodal strategies, such as physical activity and cognitive training, aimed at enhancing resilience. If broadly adopted, such proactive approaches could reduce the future burden of MCI and dementia, offering a promising pathway to alleviate the escalating societal and economic costs of neurodegenerative disease.

This study has several limitations. First, as an observational study, it cannot establish a causal relationship between MCR and incident MCI. While our findings suggest that motor dysfunction, specifically slow gait speed, plays a more prominent role in predicting MCI transition, further research is needed to definitively establish the dominance of one component over the other in the underlying pathological processes. Future multicenter cohort studies with larger sample sizes are needed to evaluate intervention strategies targeting MCR and their effectiveness in slowing dementia progression. Second, recall bias is an inherent limitation, as SCC was assessed solely through self-reported questionnaires at baseline. To mitigate this, the questionnaire wording was optimized during the development phase. Third, as a single-center cohort study, our findings may have limited generalizability. Validation in diverse populations and multicenter cohort studies is necessary to minimize selection bias and confirm the broader applicability. Finally, the one-year follow-up period, while suitable for detecting rapid transitions, may not fully capture slower, gradual progressions to MCI. This could lead to an overestimation of MCR to MCI conversion rates, and future studies with longer follow-up are needed to better characterize the natural history of MCR.

## 5 Conclusion

This study highlights MCR as a significant risk factor for MCI among community-dwelling older adults. By establishing MCR as a predictor of MCI, our findings extend dementia risk prediction to earlier stages of cognitive decline, reinforcing the importance of early screening and intervention.

## Data Availability

The original contributions presented in the study are included in the article/Supplementary material, further inquiries can be directed to the corresponding authors.
