# Supplementary material for: Association of motoric cognitive risk syndrome and incident mild cognitive impairment in community-dwelling older adults
Source: Front Neurol. 2026 Mar 20;17:1787145. doi: 10.3389/fneur.2026.1787145 (PMC13046496; doi:10.3389/fneur.2026.1787145)
Supplement: Supplementary file 1 [file Data_sheet_1.pdf]

Supplementary Material

Supplemental Figure 1: Criteria for the diagnosis of MCR and MCI

Criteria for the diagnosis of motoric cognitive risk syndrome (MCR)

(1) subjective cognitive complaint
(2) slow gait speed
(3) functionally independent
(4) absence of dementia

Criteria for the diagnosis of mild cognitive impairment (MCI)

(1) objective cognitive impairment
(2) normal general cognitive function
(3) functionally independent
(4) absence of dementia

Abbreviations: MCR, motoric cognitive risk syndrome; MCI, mild cognitive impairment.

Supplemental Table 1: Gait speed cutoff points stratified by age and sex

Age group (years)	Mean	SD	Cutoff point
Male	cm/s	cm/s	cm/s
60–74	82.60	17.49	65.11
≥ 75	80.23	18.84	61.39
Female			
60–74	81.41	18.87	62.54
≥ 75	78.66	20.70	57.96

Abbreviations: cm, centimeter; MCR, motoric cognitive risk syndrome; s, second; SD, standard deviation.

Supplemental Table 2: MMSE cutoff points for defining objective cognitive impairment in MCI

Educational level	Mean	SD	Cutoff point
Illiteracy	21.62	2.16	19.46
Elementary school	27.05	2.75	24.31
Middle school or above	28.52	1.66	26.86

Abbreviations: MCI, mild cognitive impairment; MMSE, mini-mental state examination; SD, standard deviation.

Supplemental Table 3: Criteria for the diagnosis of comorbidities

Comorbidity	Diagnostic criteria
Hypertension[1]	≥ 1 of the following:<br>- Systolic BP ≥ 140 mmHg or diastolic BP ≥ 90 mmHg<br>- Physician diagnosis in medical record<br>- Current antihypertensive therapy
Diabetes Mellitus[2]	≥ 1 of the following:<br>- Fasting glucose ≥ 7.0 mmol/L<br>- Postprandial glucose ≥ 11.1 mmol/L<br>- Hemoglobin A1c ≥ 6.5%<br>- Active pharmacotherapy (oral/insulin)
Dyslipidemia[3]	≥ 1 of the following:<br>- TC ≥ 5.2 mmol/L<br>- LDL-C ≥ 3.4 mmol/L<br>- TG ≥ 1.7 mmol/L<br>- HDL-C < 1.0 mmol/L (Male), < 1.3 mmol/L (Female)
Coronary heart disease[4]	≥ 1 of the following:<br>- Self-reported history<br>- History of PCI/CABG
Stroke[5]	≥ 1 of the following:<br>- Self-reported history<br>- Neurologist-confirmed diagnosis<br>- Neuroimaging evidence<br>- Current anti-platelet/anticoagulant regimen

Abbreviations: BP, blood pressure; TC, total cholesterol; LDL-C, low-density lipoprotein cholesterol; TG, triglycerides; HDL-C, high-density lipoprotein cholesterol; PCI, percutaneous coronary intervention; CABG, coronary artery bypass grafting.

Supplemental Table 4: Prevalence of MCR stratified by age, gender, and educational level

	Non-MCR (n)	MCR (n)	Prevalence % (95% CI)	*P*-value
Overall	725	128	15.0 (12.7 – 17.6)	-
Age, years	-	-	-	-
< 75	610	108	15.0 (12.5 – 17.9)	0.060
≥ 75	115	20	14.8 (9.3 – 21.9)	-
Sex	-	-	-	-
Male	265	47	15.1 (11.3 – 19.5)	0.348
Female	460	81	15.0 (12.1 – 18.3)	-
Education	-	-	-	-
Elementary school or below	14	2	12.5 (3.7 – 28.7)	0.990
Middle school	201	26	11.5 (7.3 – 15.6)	-
High school	260	54	17.2 (13.0 – 21.4)	-
College or above	250	46	15.4 (11.4 – 19.7)	-

Abbreviations: CI: Confidence Interval; MCR, motoric cognitive risk syndrome.

Reference

1. Mancia G, Rosei EA, Azizi M, et al. 2018 ESC/ESH Guidelines for the management of arterial hypertension.

2. ElSayed NA, Aleppo G, Aroda VR, et al. 2. Classification and Diagnosis of Diabetes: Standards of Care in Diabetes-2023. *Diabetes Care* 2023; 46: S19–S40.

3. Catapano AL, Graham I, De Backer G, et al. 2016 ESC/EAS Guidelines for the Management of Dyslipidaemias. *Rev Esp Cardiol (Engl Ed)* 2017; 70: 115.

4. Knuuti J. 2019 ESC Guidelines for the diagnosis and management of chronic coronary syndromes The Task Force for the diagnosis and management of chronic coronary syndromes of the European Society of Cardiology (ESC). *Russ J Cardiol* 2020; 25: 119–180.

5. Warner JJ, Harrington RA, Sacco RL, et al. Guidelines for the Early Management of Patients With Acute Ischemic Stroke: 2019 Update to the 2018 Guidelines for the Early Management of Acute Ischemic Stroke. *Stroke* 2019; 50: 3331–3332.
